# Insights into mutualism mechanism and versatile metabolism of *Ketogulonicigenium vulgare* Hbe602 based on comparative genomics and metabolomics studies

**DOI:** 10.1038/srep23068

**Published:** 2016-03-16

**Authors:** Nan Jia, Ming-Zhu Ding, Jin Du, Cai-Hui Pan, Geng Tian, Ji-Dong Lang, Jian-Huo Fang, Feng Gao, Ying-Jin Yuan

**Affiliations:** 1 Key Laboratory of Systems Bioengineering (Ministry of Education), School of Chemical Engineering and Technology, Tianjin University, Tianjin, 300072, PR China; 2 SynBio Research Platform, Collaborative Innovation Centre of Chemical Science and Engineering (Tianjin), School of Chemical Engineering and Technology, Tianjin University, Tianjin, 300072, PR China; 3 Sequencing platform of Tsinghua University, Beijing, 100084, PR China; 4 Department of Physics, Tianjin University, Tianjin, 300072, PR China

## Abstract

*Ketogulonicigenium vulgare* has been widely used in vitamin C two steps fermentation and requires companion strain for optimal growth. However, the understanding of *K. vulgare* as well as its companion strain is still preliminary. Here, the complete genome of *K. vulgare* Hbe602 was deciphered to provide insight into the symbiosis mechanism and the versatile metabolism. *K. vulgare* contains the LuxR family proteins, chemokine proteins, flagellar structure proteins, peptides and transporters for symbiosis consortium. Besides, the growth state and metabolite variation of *K. vulgare* were observed when five carbohydrates (D-sorbitol, L-sorbose, D-glucose, D-fructose and D-mannitol) were used as carbon source. The growth increased by 40.72% and 62.97% respectively when *K. vulgare* was cultured on D-mannitol/D-sorbitol than on L-sorbose. The insufficient metabolism of carbohydrates, amino acids and vitamins is the main reason for the slow growth of *K. vulgare.* The combined analysis of genomics and metabolomics indicated that TCA cycle, amino acid and nucleotide metabolism were significantly up-regulated when *K. vulgare* was cultured on the D-mannitol/D-sorbitol, which facilitated the better growth. The present study would be helpful to further understand its metabolic structure and guide the engineering transformation.

*K. vulgare* was identified as a member of the *Proteobacteria*^1^, which can convert L-sorbose to the precursor of vitamin C, 2-keto-L-gulonic acid (2-KGA)^2^. Currently, the genomes of two typical strains in the genus *K. vulgare* have been sequenced completely^3^,^4^. However, the understanding of *K. vulgare* is still preliminary partly because of its unique nature. It was found that the *K. vulgare* mono-culture system grew poorly, even on rich natural media such as yeast extracts, peptones and corn steep liquor. The *Bacillus* species are usually co-cultivated with *K. vulgare* to achieve a better growth rate of *K. vulgare* and an adequate 2-KGA yield^5^. Addition of amino acids^3^, vitamins^6^ and glutathione^7^ could enhance the growth of *K. vulgare*, implying the defects of sulfur and oxidation metabolism, vitamin and amino acid synthesis. However, further research is needed to know the reason for the weak growth of *K. vulgare* and how to adapt its growth state in symbiosis system.

Using GC-TOF/MS technology, metabolomics was suitable for analyzing the variations in primary metabolites, such as intermediates in central carbon metabolism and amino acid biosynthesis. The metabolomics approach has been demonstrated in our previous studies on the interactions between *K. vulgare* and *Bacillus* strain^8^. Here we combine comparative genomics and metabolomics to explore the mechanism behind the positive effects of different carbon sources. Previous research showed that heterologously expression of folate biosynthesis from *Lactococcus lactis* in *K. vulgare* could enhance the growth and 2-KGA production^9^, and the present study would be helpful for further analysis of the metabolism network and identification of the genetic targets for strain improvement.

## Results

### General genomic properties of *K. vulgare* Hbe602

**Figure 1:**
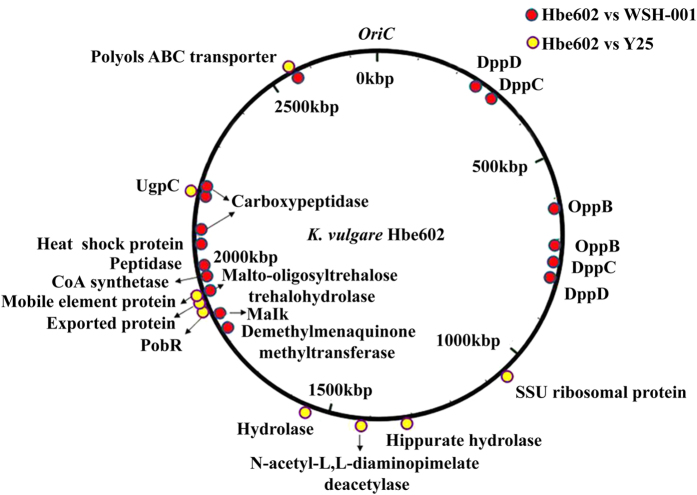
Comparative genomic analysis of the difference among *K. vulgare* Hbe602, WSH-001 and Y25. The chromosome of *K. vulgare* Hbe602 was used in comparison, the differences with WSH-001 and Y25 were labeled in the red dots and yellow dots, respectively.

The genome of *K. vulgare* Hbe602 consists of one circular chromosome and two plasmids, which encodes 3,178 predicted proteins, 58 tRNAs and 15 rRNAs in total (Fig. S1 and Table S1). For the circular chromosome, the predicted replication origin is located at 2,765,502-281 nt, which is closely next a *maf* gene (282-887 nt) encoding septum formation protein Maf. The analysis of replication origins for bacteria in DoriC, a database of *oriC* regions in bacterial and archaeal genomes^10^,^11^, has also shown the feature that *oriC* region is adjacent to a *maf* gene is conserved in the family *Rhodobacteraceae*. For the plasmids, the putative origins of replication are also at 0 kb, and both contain repeat sequences. Phylogenetic analysis of *K. vulgare* Hbe602 with other species could provide putative evolutionary histories and phenotypic diversity (Fig. S2). To date, *K. vulgare* Hbe602 revealed the high homogeneity with other *K. vulgare* species (Y25 and WSH-001), which was related closely to *Rhodobacter* and *Paracoccus*. Furthermore, the genome-scale sequence comparison by LAST software available at http://last.cbrc.jp shows the genome similarity between *K. vulgare* Hbe602 with WSH-001 is higher than that with Y25 (Fig. S3)^12^. Through the whole genome comparison, the different genes with the nucleotide identities lower than 90% are obtained, mostly focused on proteolytic enzymes and transporters (Fig. 1). Previously, *K. vulgare* was mistakenly regarded as a member of *Gluconobacter oxydans* due to the similar phenotype^1^,^13^. To facilitate the gene function analysis, *G. oxydans* 621H, *Escherichia coli* K-12 and *R. sphaeroides* 2.4.1 are selected to compare the distribution of COG classification with *K. vulgare* Hbe602 (Fig. S4 and Table S2). Consequently, in *K. vulgare*, the number of genes related to amino acid transport and metabolism (E) is similar to those in *E. coli* and *R. sphaeroides* and higher than that in *G. oxydans*. Those functional genes encode many transporters, which absorb the nutrients to compensate its metabolic defect. Besides, the number of transcriptional regulation related genes (K) is even more than that in *E. coli*. The strong transcriptional regulation ability may be activated to form a symbiotic relationship with other microorganisms. With the aid of KEGG analysis, metabolic network is obtained, including the carbohydrate-active enzymes, nitrogen and central carbon metabolism, amino acid and cofactor metabolism.

### Symbiosis mechanism related genes

The quorum sensing phenomenon has been well established in Gram-negative bacteria, where N-acyl homoserine lactones are the diffusible communication molecules that modulate cell-density-dependent phenotypes^14^. Genomic analysis of 265 *Proteobacteria* species shows that at least 68 species contain LuxI (AHL synthase) and LuxR (transcriptional activator), while 45 species only contain LuxR^15^. *K. vulgare* only contains LuxR and lacks LuxI, so it may produce AHL by other means, or obtains the AHL signaling molecules from other microorganisms^16^. The LuxR family protein is the hub of quorum sensing system, which regulates many kinds of proteins and thus affects the global quorum sensing. *K. vulgare* also contains phosphorylated protein CtrA taking part in flagella mobile and cell division, which is widely present in the alpha-*Proteobacteria*^17^. Besides, *K. vulgare* contains two HK97 family proteases, which are generally encoded by phage. They were also found in *R. capsulatus* as gene transfer agent, which can mediate gene transfer^18^. Since *K. vulgare* and *R. capsulatus* are the neighbor strains, the HK97 family protease in *K. vulgare* may not be used for the degradation of protein, but the gene transfer agent. Furthermore, *K. vulgare* Hbe602 contains several chemotaxis proteins (Table S3), including a Che cluster of CheA, CheB, CheD, CheR, CheW and CheX, to respond to the environmental variation. We also found a large cluster with 37 flagella-related genes (Table S4), which operated flagella structure synthesis and flagella motility. The ability of chemotaxis and motility in *K. vulgare* Hbe602 is connected with its natural soil environment, and is used for inducing the optimal symbiotic relationship. Moreover, *K. vulgare* Hbe602 encodes 18 predicted proteases, 48 peptides and almost 380 transporter related proteins, which can effectively decompose the related substances from the cultured environment or companion strain^19^.

### Metabolomics analysis of the versatile metabolism in *K. vulgare* Hbe602

**Figure 2:**
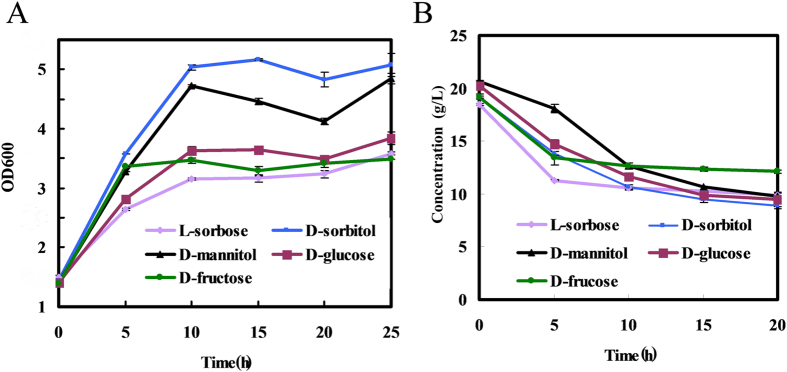
Growth state of *K. vulgare* Hbe602 in different carbon sources. **(A**) Growth curve the *K. vulgare* Hbe602 strain grown in seed medium with different carbon source (L-sorbose, D-sorbitol, D-mannitol, D-glucose and D-fructose). The Y axis represents the average OD_600nm_ at each time point; (**B**) Extracellular concentration of different carbohydrates.

**Figure 3:**
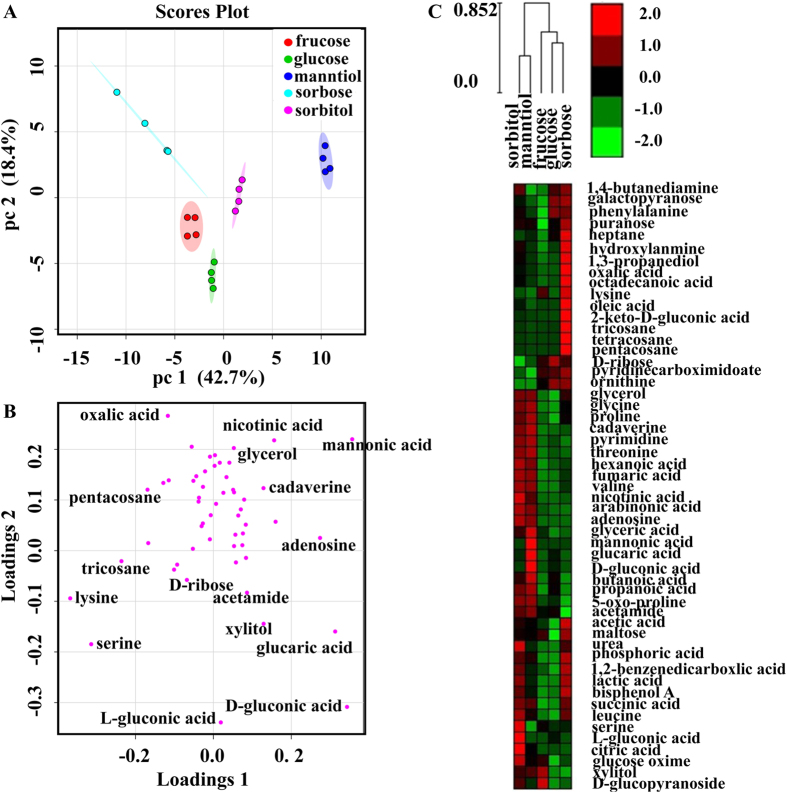
Statistics analysis of the metabolite distribution among different samples. **(A**) Scores plot of the samples; (**B**) Loading plot of the samples; (**C**) Heat map of metabolite expressions in different samples.

Due to the limited understanding of genome and metabolic network, the role of L-sorbose in the bio-conversion for *K. vulgare* is not clear. We try to add different carbon sources (L-sorbose, D-sorbitol, D-mannitol, D-fructose and D-glucose) for observing the growth phenotype and analyzing the key genes. The growth conditions of *K. vulgare* varied dramatically on five carbon sources and the highest growth value with D-mannitol was about 1.63 times than that with L-sorbose (Fig. 2). Metbolomics analysis was carried out to compare the metabolome of *K. vulgare* cultured on five carbon sources. A total of 54 metabolites were identified, involving the central carbon metabolism, amino acid and lipid biosynthesis. Multivariate data analysis was used to identify the bio-markers in response to the environment changes. The cells cultured in sorbitol and mannitol were clustered together at a short distance from those cultured in sorbose (Fig. 3A,C). They may have the similar way to improve the growth of *K. vulgare*. From the distances of the metabolites away from the origin in PCA loading plot, many metabolites affecting primary metabolism were responsible for the differences in the samples, including serine, lysine, nicotinic acid, 2-keto-D-gluconic acid, xylitol and mannonic acid (Fig. 3B). Fifty-four metabolites were categorized into 4 clusters based on the variable similarity patterns (Fig. S5). Twenty-two metabolites (clusters 1 and 4) maintained at a higher level in the cells cultured in sorbitol/manntiol than those in sorbose. Ten metabolites (cluster 2) accumulated mostly in sorbose and 7 metabolites (cluster 3) accumulated mostly in sorbitol.

**Figure 4:**
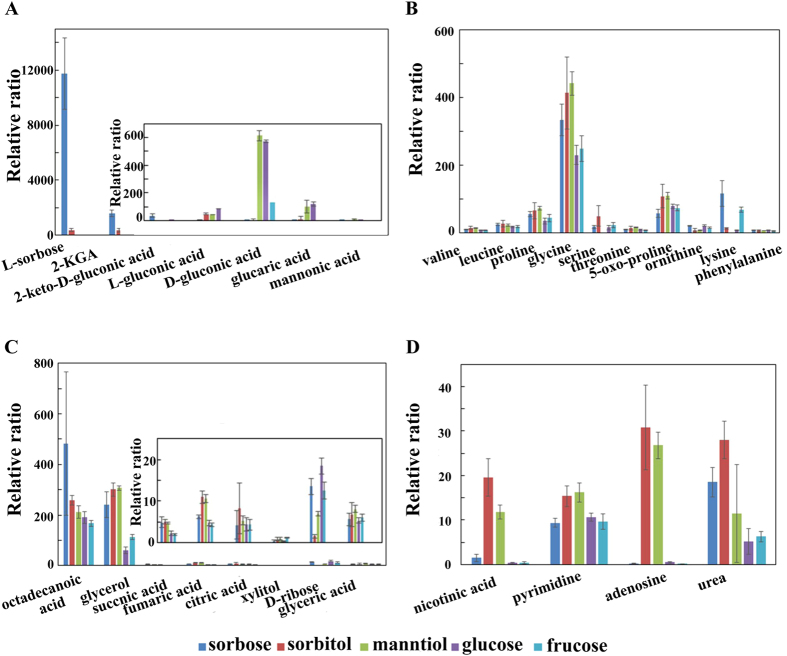
Changes of detected intermediates in different carbon sources. **(A**) Related acid production; (**B**) Glycolysis and tricarboxylic acid cycle; (**C**) Amino acids; (**D**) Others. The relative abundance was calculated by normalization of peak area of each metabolite to internal standard, and the error bars showed the standard deviations of four replicates.

It is interesting to remark that *K. vulgare* Hbe602 preformed a better growth on seed medium supplied with D-sorbitol/D-mannitol compared with L-sorbose. Actually, sorbose and 2-KGA were detected in the cells when sorbitol was used as substrate, and mannoic acid was detected when mannitol was used as substrate (Fig. 4A). *G. oxydans* could convert D-glucose to 2-/5-keto-gluconic acid^20^. We observed the related acid product when *K. vulgare* was cultured in glucose, fructose and mannitol as well (Fig. 4A). *K. vulgare* lacks many amino acid synthesis pathways (Fig. S6) and we only found 10 amino acids in the cells (Fig. 4B). Among them, valine, leucine, proline, glycine, threonine and 5-oxo-proline were biomass relevant, which maintained at a higher level in the cells cultured in sorbitol/manntiol compared with those in sorbose. Serine was maintained at a significant high level when cells cultivated in sorbitol. Glycine, proline, serine and threonine could be converted into intermediates of the tricarboxylic acid (TCA) cycle and used for generating energy or other components. Besides, the levels of valine, proline, glycine and serine were extremely higher in the cells receiving GSH^21^. Supplement of glycine, proline and threonine led to 29.1%, 24.9% and 28.1% increased in the growth of *K. vulgare*, respectively^3^. In addition, proline has acted as osmolarity protective agent, which greatly accumulated in sorbitol/manntiol for anti-stress protection^22^. Four important intermediates in TCA, including citric acid, octadecanoic acid, succinic acid and fumaric acid were identified, and maintained higher levels in the cells cultured in sorbitol/manntiol than those in sorbose (Fig. 4C). The urea concentration in the cells cultured in sorbitol was 1.5 times of those in sorbose, and nicotinic acid, pyrimidine and adenosine represented higher concentration when the cells cultured in sorbitol/manntiol than in sorbose (Fig. 4D).

## Discussion

It has always been an important subject for enhancing the growth ability of *K. vulgare*. In the present study, the metabolic network of *K. vulgare* was obtained by KEGG analysis, and the environmental response in different carbon sources (L-sorbose, D-sorbitol, D-mannitol, D-fructose and D-glucose) was analyzed by metabolomics studies. The genomics analysis found that *K. vulgare* lacks the endoenzyme, which transforms D-mannitol, L-sorbose, D-sorbitol and D-glucose to D-frucose (Fig. S7). The different sugars may be obtained through a large number of trans-membrane proteins, and different combinations of L-sorbose dehydrogenases and L-sorbosone dehydrogenases from *K. vulgare* were introduced into *G. oxydans* to converse L-sorbose to 2-KGA^23^. Additionally, the digestive enzymes of glycoside were found, such as glycoside hydrolase, beta-glucosidase and glycosyl hydrolase, involving in the substance utilization.

In the nitrogen metabolism of *K. vulgare*, we found the urease and four urea transporters, which can hydrolyze urea and release ammonia for re-utilizing. Besides, we also found the ammonia transporter and carbamoyl-phosphate synthase for absorbing foreign ammonia, and nitrogenase for nitrogen fixation. The nitrogen metabolism regulated protein NtrC, NtrY and NtrX were encoded by a gene cluster. NtrC combined with the promoter region of *glnB* to activate *gln*B/*gln*A under nitrogen-deficient conditions^24^ and regulated the *ntrY* and *ntrX*^25^. The urea concentration within the cells cultured in sorbitol was 1.5 times of those in sorbose medium, providing enough substrate for amino synthesis and pH adjustment^26^.

In the central carbon metabolism of *K. vulgare*, we identified the complete TCA cycle pathway and pentose phosphate pathway. Twelve C4-dicarboxylate transporters used ionic electrochemical potential gradient to absorb C4-dicarboxylic acid substances to compensate the TCA cycle^27^. The key gene encoding 6-phosphofruco-2-kinase is absent in the glycolytic pathway. Therefore, the pentose phosphate pathway (PPP) is used as the major central carbohydrate metabolism pathway to link up the TCA cycle.

In the cofactor and nucleotide metabolism of *K. vulgare*, metabolism pathway of thiamine, biotin and porphyrin, biosynthesis pathway of folate and ubiquinone are not complete. The companion strain has complete B-family vitamin synthesis pathways, which can complement *K. vulgare*^19^. The cluster *pqqABCDEN* of the pyrroloquinoline quinone (PQQ) biosynthesis pathway was isolated and over-expressed in *K. vulgare* Hbe602^28^. The *K. vulgare* mutant harboring a plasmid with the complete *pqqABCDE* cluster achieved 3-folds higher level of PQQ biosynthesis than the wild-type. PqqN was annotated as a channel protein based on the best blast hit with the bestrophin in *Shinella* strain^29^, and co-expression of *pqqN* and *pqqABCDE* decreased the PQQ level^28^. Furthermore, the cell lysis of companion strain provided purine for *K. vulgare*, and the addition of purine improved growth and 2-KGA production of *K. vulgare*^30^. From the proteomic study of experimental evolution in previous study, we found the biosynthesis of purine and pyrimidine was improved in the evolved *K. vulgare* in the co-culture^31^. Nicotinic acid, pyrimidine and adenosine were really high concentration in the cultured cells of sorbitol/manntiol than those of sorbose, which could be used as an alternative source to participate or adjust the related metabolic reactions^32^.

In the amino acid metabolism of *K. vulgare*, three reactions during the conversion of succinate to glutamic acid are defective. In the methionine cycle, *K. vulgare* converses methionine to S-Adenosylmethionine (SAM), and then transforms to S-adenosyl homocysteine cysteine and homocysteine, again into methionine. The last step of the methionine cycle reaction need N5-methyl-tetrahydrofolate as the methyl transfer agent, while tetrahydrofolic acid synthetic pathway is defective in *K. vulgare*, which may stop the normal circulation^33^. In the tryptophan metabolism pathway, *K. vulgare* only contains some isolated reactions and the conversion steps for tryptophan to acetyl CoA/FAD/FADH_2_ are absent. Besides, *K. vulgare* is absent of the fatty acid metabolism pathway so that it can’t obtain acetyl CoA directly. The concentration of acetyl CoA significantly contributed to the formation of citric acid in TCA cycle, which can be used not only as an electron acceptor, but also as a precursor for amino acid biosynthesis and ATP production. Hence, the lack of tryptophan metabolism pathway severely affected the growth of *K. vulgare*. Furthermore, *K. vulgare* harbores the complete valine, leucine and isoleucine biosynthesis pathways, but only contains several isolated reactions in the related degradation pathway. The lack of degradation ability may lead to the amino acid accumulation in *K. vulgare*, providing extracellular amino acids to companion strain in consortium^34^.

## Methods

### Cultivation and DNA extraction

The *K. vulgare* Hbe602 strain was cultured in 250 mL flasks with 50 mL of seed medium (30 °C, 250 rpm) supplied with 2% carbon source for 35 h. The seed medium contains 3 g/L beef extract, 3 g/L yeast powder, 3 g/L corn steep liquor, 0.2 g/L MgSO_4_, 1 g/L KH_2_PO_4_, 1 g/L urea and 10 g/L peptone. The genome sample was carried out by CTAB/NaCl extraction^35^. The quality of the DNA was assessed by spectrophotometer and gel electrophoresis. DNA samples with a 260/280 nm absorbance ratio of 1.8–2.0 and a 260/230 nm absorbance ratio of 2.0–2.2 were considered pure and then used for the library construction and sequencing.

### Determination of 2-KGA and different substrates

2-KGA and different substrates were detected by HPLC using an Aminex HPX-87H column (Bio-Rad, Hercules, CA, USA) coupled with a refractive index (RI) detector. 5 mM H_2_SO_4_ was used as the mobile phase at a speed of 0.6 mL/min and the RI temperature was kept at 65 °C.

### Sequencing and assembly

Using the 454 GS FLX system, single-end libraries with 15-fold coverage and mate paired-end libraries with 10-fold coverage were constructed. The genome was sequenced by the Sanger shotgun approach. The reads were assembled into 6 contigs by using the 454 Newbler assembler and the gaps between the contigs were closed by PCR amplification.

### Genome annotation and bioinformatics analysis

The genome analysis was carried out by the Rapid Annotation using Subsystems Technology (RAST) analysis platform^36^. The function of genes was also annotated by using BLAST^37^ against Kyoto Encyclopedia of Genes and Genomes (KEGG) database^38^ and Clusters of Orthologous Groups of proteins (COG) database^39^. The tRNAs and rRNAs were predicted by tRNAscan-SE^40^ and RNAmmer^41^. The subcellular location of proteins and the signal peptides were predicted by PSORT^42^ and SignalP 4.0^43^. The origin of replication (*oriC*) and putative DnaA boxes were identified by Ori-Finder^44^. CVTree was performed for the phylogenetic analysis^45^ and the phylogenetic tree was generated using the MEGA program^46^. The GC-Profile was used to compute the GC content variation in DNA sequences and to predict the horizontal gene transfer^47^. CGView Server was used for the visualization of circular genomes^48^ and the metabolic network was constructed by KEGG automatic annotation server KAAS^49^.

### Nucleotide sequence accession numbers

The sequence of the *K. vulgare* Hbe602 genome has been deposited at DDBJ/EMBL/GenBank under the accession numbers CP012908, CP012909 and CP012910.

### Metabolites extraction and derivatization

Cells cultured in different carbon source of logarithmic growth phase were quenched and extracted as intracellular metabolites according to our previous method^50^. An extra group of quenched cells was washed and dried to calculate the dry weight of the sampled cells. The 10 μL succinic *d*_4_ acid (0.1 mg/mL) was used as an internal standard to correct for minor variations occurring during sample preparation and analysis. The extracts of intracellular were lyophilized and four independent experiments were performed for each sample. Two-stage chemical derivatization was performed as described previously^50^. Firstly, methoximation of the carbonyl groups was carried out by dissolving sample in 50 μL methoxamine hydrochloride (20 mg/mL in pyridine) and incubating it at 40 °C for 60 min. Then, 80 μL N-methyl-N-(trimethylsilyl) trifluoroacetamide (MSTFA) was added and it was incubated at 37 °C for 30 min for trimethylsilylation.

### Metabolomic analysis by GC-TOF/MS

Metabolites were analyzed by GC-TOF/MS (Waters Corp., USA) as described previously^50^. The 1 μL derivatized sample was injected by Agilent 7683 autosampler into GC (Agilent 6890) which was equipped with DB-5MS column (30 m × 0.25 mm × 0.25 μm, J&W Scientific, Folsom, CA). The oven temperature was programmed as: 70 °C for 2 min, then increased to 290 °C (5 °C/min), holding for 3 min. The ion source temperature and ionization current were 250 °C and 40 μA, respectively. The mass scan range was 50–800 m/z. Peak detection, deconvolution, and peak quantification were performed using Masslynx software 4.1^51^. Metabolites were identified by comparing their mass fragmentation patterns with NIST mass spectral library^52^. The area of each acquired peak was normalized against the internal standard and dry cell weight for calculating the relative abundance. Multivariate data analysis was preformed by principal-components analysis (PCA)^53^ and hierarchical cluster analysis (HCA)^54^ to view the relative differences in the metabolites concentrations among diverse conditions.

## Additional Information

**How to cite this article**: Jia, N. *et al.* Insights into mutualism mechanism and versatile metabolism of *Ketogulonicigenium vulgare* Hbe602 based on comparative genomics and metabolomics studies. *Sci. Rep.* **6**, 23068; doi: 10.1038/srep23068 (2016).

## Supplementary Material

Supplementary Information
