# Supplementary material for: Insights into mutualism mechanism and versatile metabolism of Ketogulonicigenium vulgare Hbe602 based on comparative genomics and metabolomics studies
Source: Sci Rep. 2016 Mar 16;6:23068. doi: 10.1038/srep23068 (PMC4793288; doi:10.1038/srep23068)
Supplement: Supplementary Information [file srep23068-s1.pdf]

SUPPLEMENTARY INFORMATION

Insights into mutualism mechanism and versatile metabolism of *Ketogulonicigenium vulgare* Hbe602 based on comparative genomics and metabolomics studies

Nan Jia, Ming-Zhu Ding*, Jin Du, Cai-Hui Pan, Geng Tian, Ji-Dong Lang, Jian-Huo Fang, Feng Gao*, Ying-Jin Yuan

* Corresponding authors: Ming-Zhu Ding, Feng Gao

Email: mzding@tju.edu.cn, fgao@tju.edu.cn

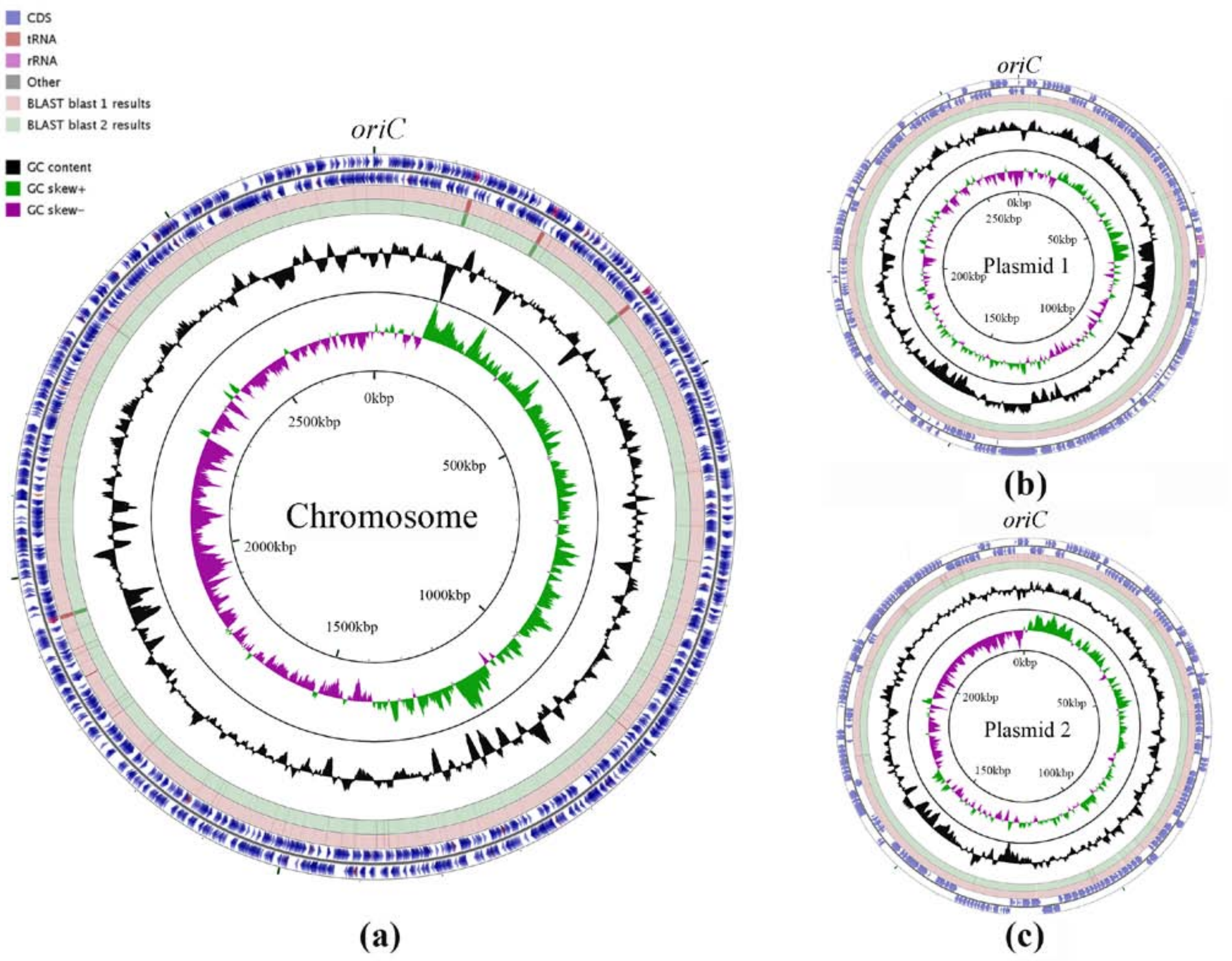

Figure S1. Circular representation of the *K. vulgare* Hbe602 genome, including (a) chromosome, (b) plasmid 1 and (c) plasmid 2. Circles 1and 2 (from the outside to the inside) show the positions of protein-coding genes (blue), tRNA genes (red) and rRNA genes (pink) on the positive and negative strands. The sequence comparison of *K. vulgare* Hbe602 against *K. vulgare* Y25 and *K. vulgare* WSH-001 are shown in circles 3 and 4. Circles 5 and 6 show plots of GC content and GC skew plotted as the deviation from the average for the entire sequence.

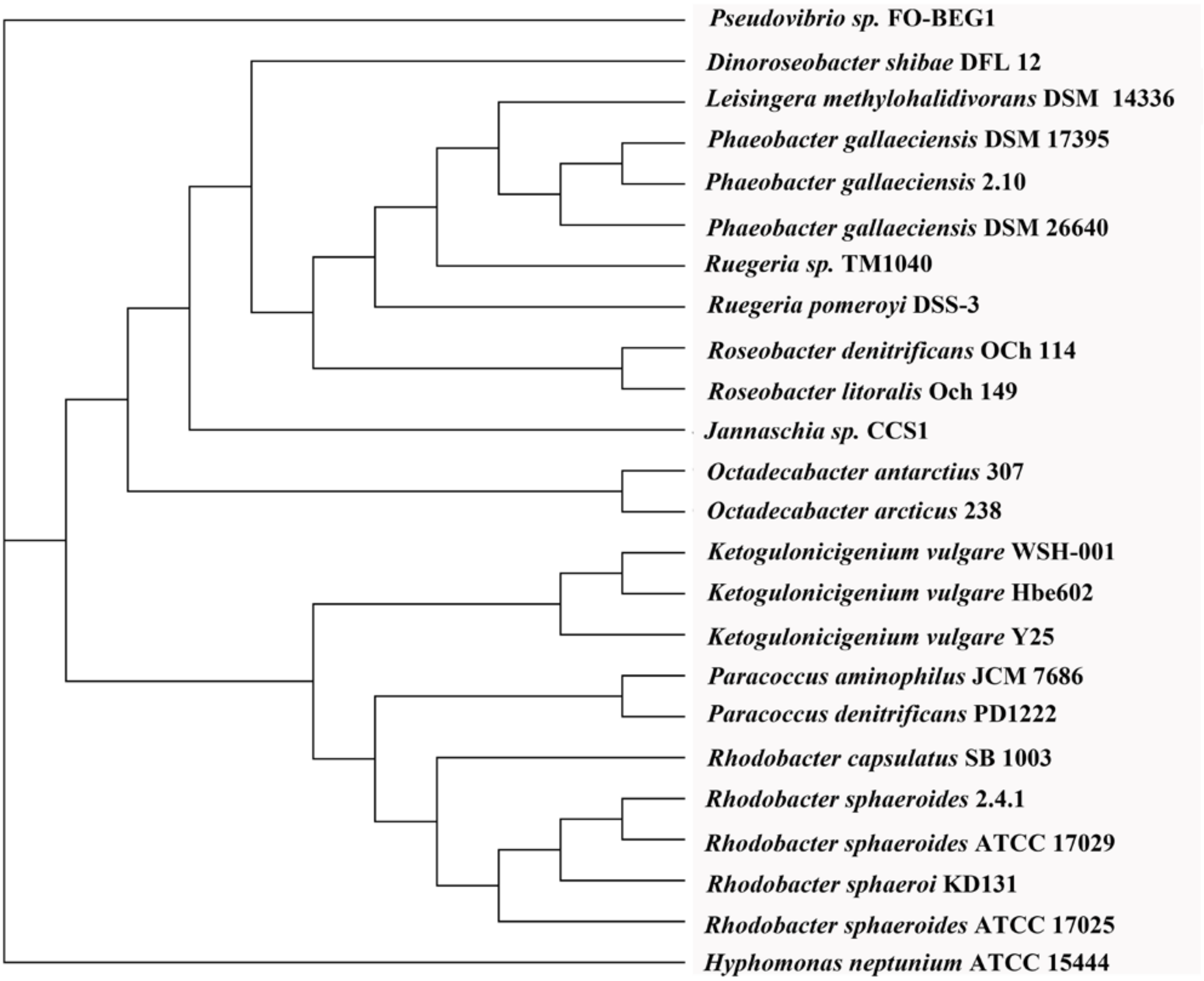

Figure S2. Phylogenetic analysis of *K. vulgare* Hbe602 with other species. The phylogenetic tree of *K. vulgare* Hbe602 was constructed using CVTree with parameters K=6 and Type=aa. The neighbor-joining tree was constructed using the MEGA5 program. Note that *Hyphomonas neptunium* ATCC 15444 was included as an outgroup.

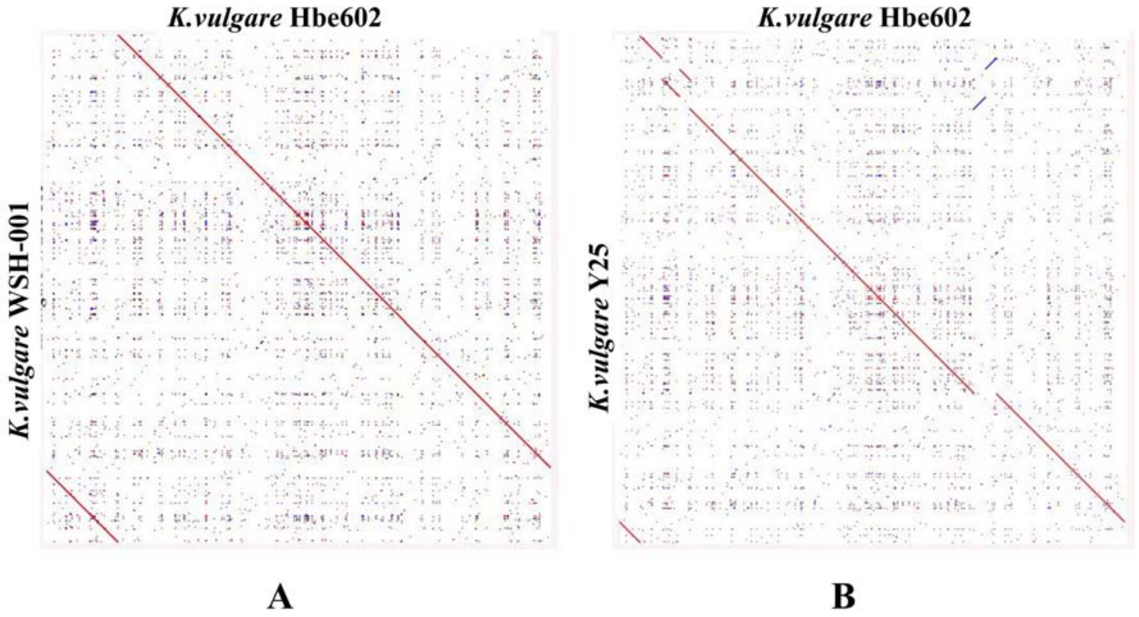

Figure S3. Genome similarity comparison between *K. vulgare* Hbe602 with WSH-001 and Y25.

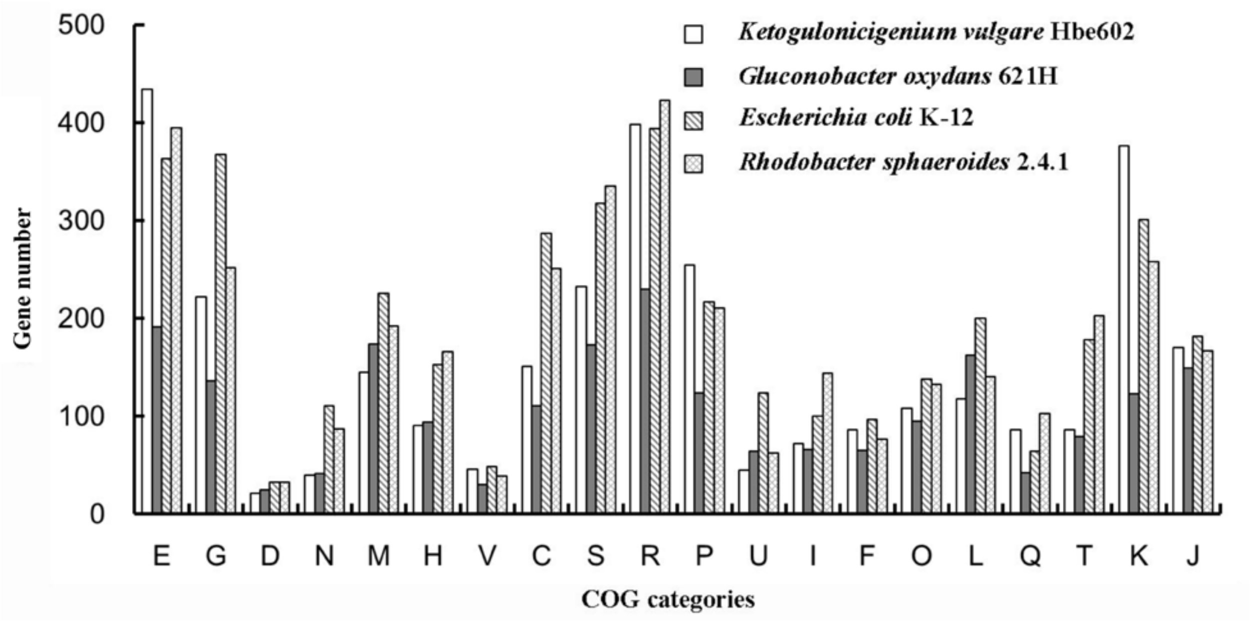

Figure S4. COG analysis of *K. vulgare* Hbe602 with other Gram-negative bacteria. Abbreviation and function description are shown as follows: D, Cell division and chromosome partitioning; M, Cell envelope biogenesis; N, Cell motility and secretion; V, Defense mechanisms; U, Intracellular trafficking and secretion; O, Posttranslational modification, protein turnover and chaperones; T, Signal transduction mechanisms; L, DNA replication, recombination and repair; K, Transcription; J, Translation, ribosomal structure and biogenesis; E, Amino acid transport and metabolism; G, Carbohydrate transport and metabolism; H, Coenzyme metabolism; C, Energy production and conversion; P, Inorganic ion transport and metabolism; I, Lipid metabolism; F, Nucleotide transport and metabolism; Q, Secondary metabolites biosynthesis, transport and catabolism; S, Function unknown; R, General function prediction only.

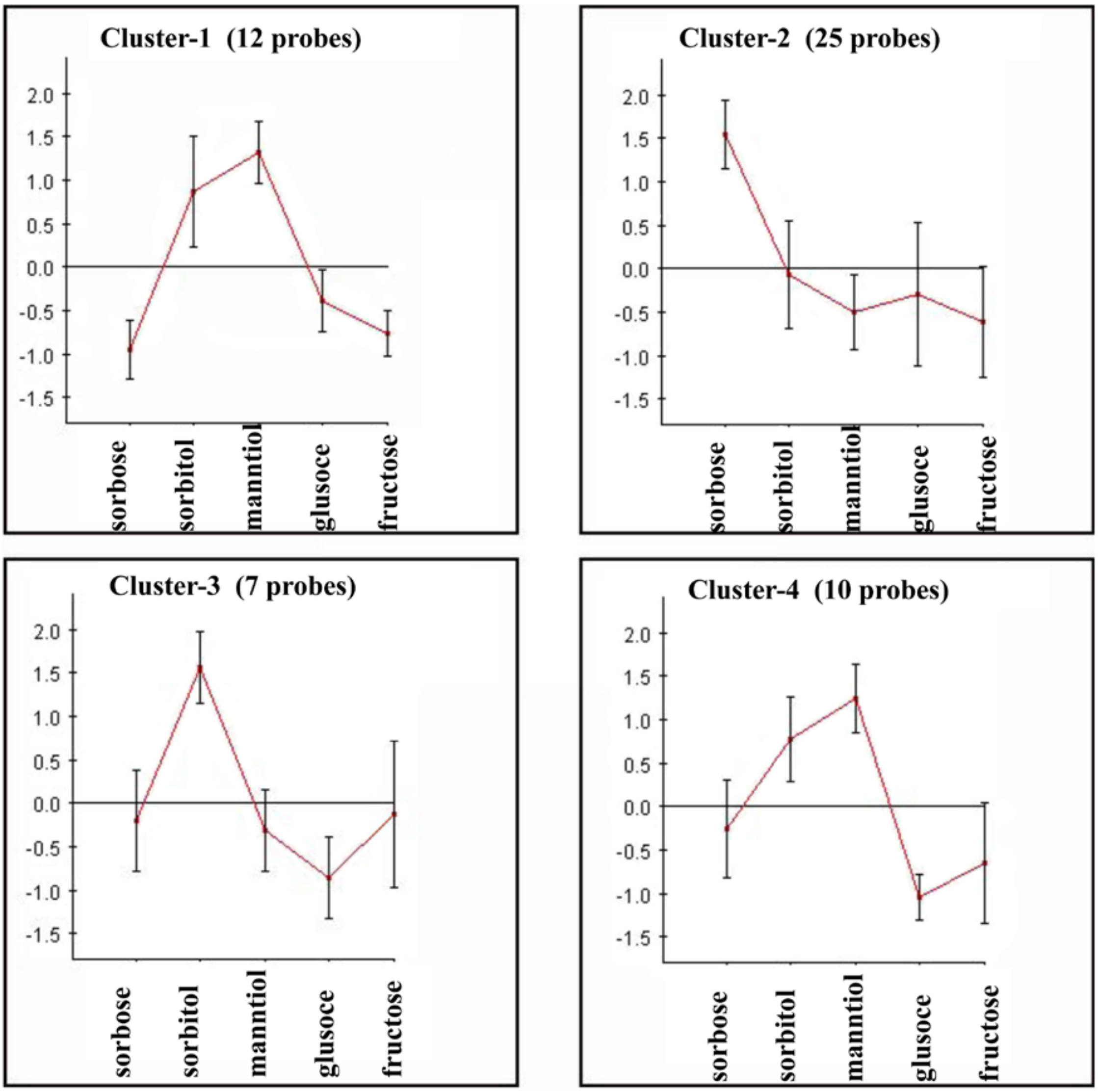

Figure S5. Hierarchy cluster analysis of metabolites. 54 metabolites were categorized into 4 clusters based on expression levels using K-means algorithm with the software Expander 4.1.

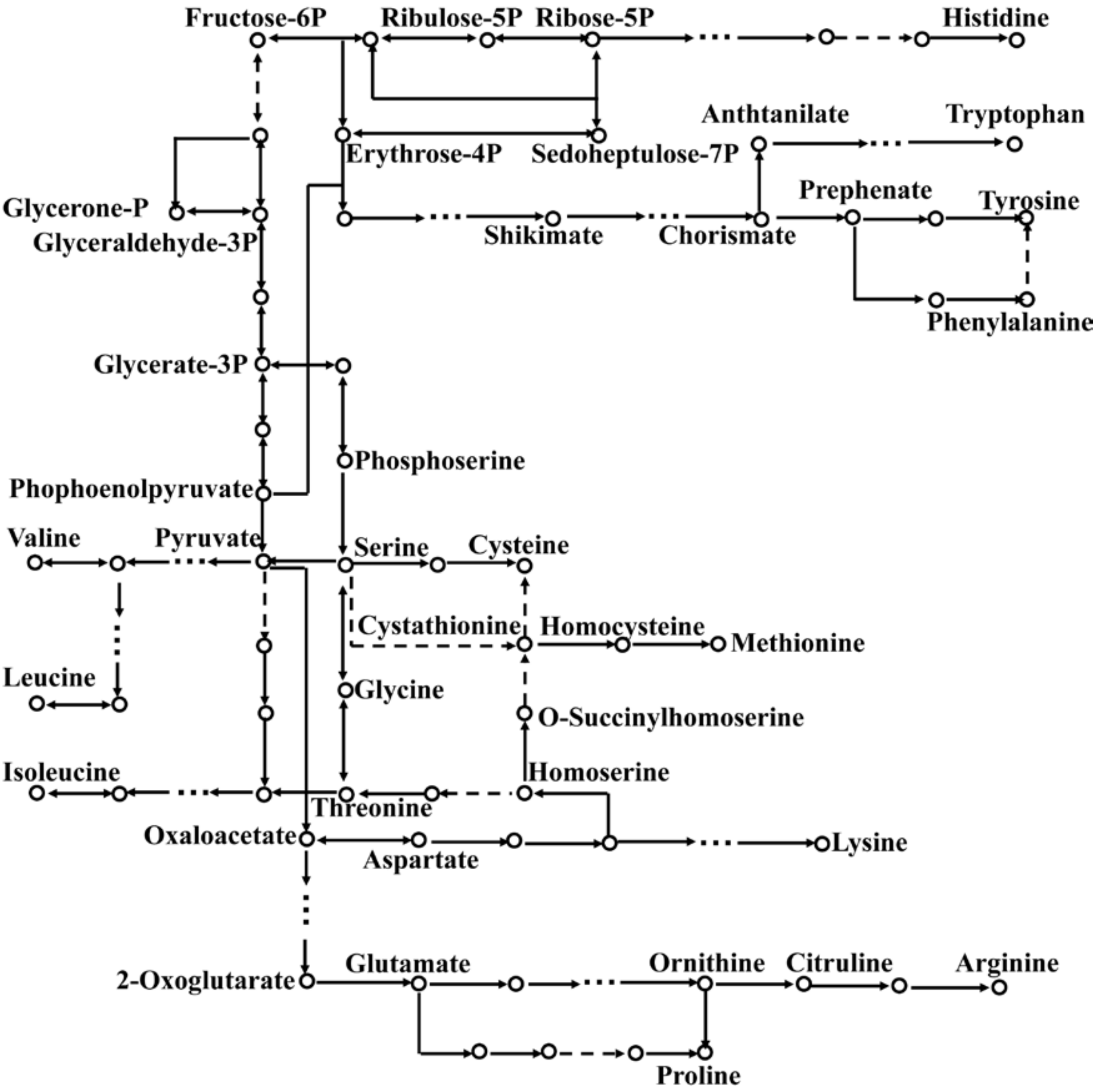

Figure S6. The amino acid synthesis network in *K. vulgare* Hbe602. The dashed line represents the defected pathways in *K. vulgare* Hbe602.

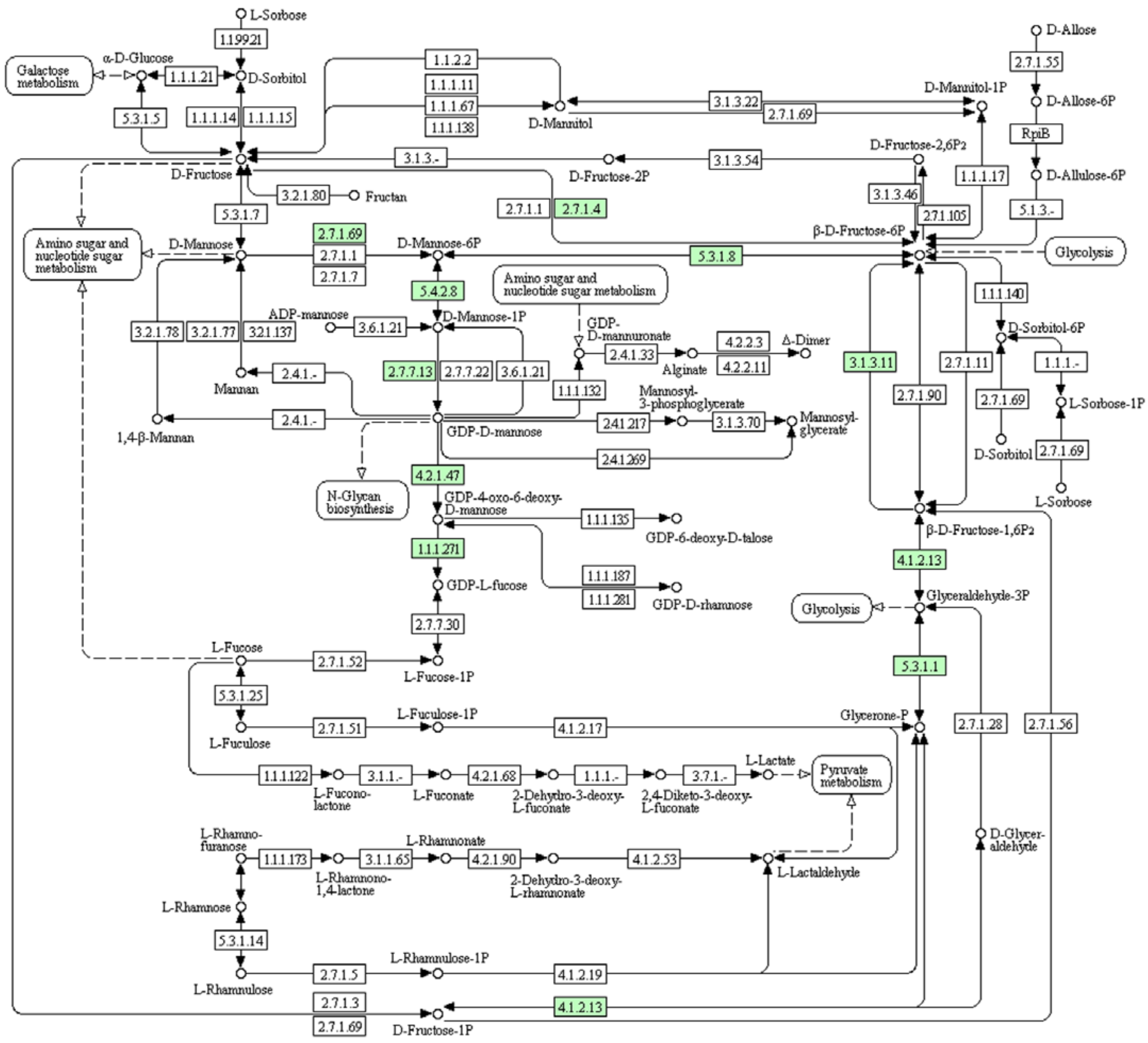

Figure S7. The monose transform related endoenzyme in *K. vulgare* Hbe602.

Table S1. General features of *K. vulgare* Hbe602 genome.

Genomic features	Chromosome	Plasmid 1	Plasmid 2
Length (bp)	2,765,686	267,988	242,716
G+C (%)	61.7	61.3	62.6
CDS	2721	243	214
tRNA	58	0	0
sRNA	12	3	0

Table S2. Proteins of *K. vulgare* Hbe602 in each COG category.

COG category	Chromosome	Plasmid1	Plasmid2
[J] Translation, ribosomal structure and biogenesis	166	3	1
[K] Transcription	302	44	30
[L] Replication, recombination and repair	105	9	4
[D]Cell cycle control, cell division,chromosome partitioning	17	3	1
[V] Defense mechanisms	34	6	6
[T] Signal transduction mechanisms	69	6	11
[M] Cell wall/membrane/envelope biogenesis	138	5	2
[N] Cell motility	36	3	1
[U] Intracellular trafficking, secretion, and vesicular transport	35	10	0
[O] Posttranslational modification, protein turnover, chaperones	103	3	2
[C] Energy production and conversion	133	10	8
[G] Carbohydrate transport and metabolism	193	14	15
[E] Amino acid transport and metabolism	333	37	64
[F] Nucleotide transport and metabolism	86	0	0
[H] Coenzyme transport and metabolism	80	7	3
[I] Lipid transport and metabolism	63	3	6
[P] Inorganic ion transport and metabolism	200	26	28
[Q] Secondary metabolites biosynthesis, transport and catabolism	64	5	17
[R] General function prediction only	347	18	33
[S] Function unknown	207	14	11

Table S3. The predicted chemotaxis proteins in *K. vulgare* Hbe602.

Locus	Annotation
KVH_6200	methyl-accepting chemotaxis protein: methyltransferase CheR
KVH_11050	methyl-accepting chemotaxis protein: signaling domain protein
KVH_08585	methyl-accepting chemotaxis protein: chemoreceptor McpA
KVH_08590	CheD family protein
KVH_08595	CheB methylesterase family protein
KVH_08600	CheX protein
KVH_08605	chemotaxis two-component response regulator protein
KVH_08610	CheA signal transduction histidine kinase
KVH_08615	chemotaxis protein CheW
KVH_08620	CheR methyltransferase

Table S4. The predicted flagella genes in *K. vulgare* Hbe602.

Locus	Annotation	Function
KVH_12265	flagella hook capping family protein	flagella structure
KVH_12270	flagella hook-length control family protein	flagella structure
KVH_12275	flagella protein FlgJ	flagella structure
KVH_12280	FlgN-like protein	flagella structure
KVH_12285	flagellin FljK (25 kDa flagellin)	regulator
KVH_12290	protein FlaF	regulator
KVH_12295	flagella protein FlbT family protein	regulator
KVH_12300	flagella protein, putative	unknown
KVH_12305	flagellum-specific ATP synthase	protein export ATPase
KVH_12310	flagella basal body rod protein FlgB	flagella structure
KVH_12315	flagella basal-body rod protein FlgC	flagella structure
KVH_12320	flagella hook-basal body protein FliE	flagella structure
KVH_12325	flagella biosynthetic protein FliQ	export apparatus
KVH_12330	flagella basal-body rod protein FlgF, putative	flagella structure
KVH_12335	flagella basal-body rod protein FlgG	flagella structure
KVH_12340	flagella basal body P-ring formation protein FlgA	flagella structure
KVH_12345	flagella L-ring protein FlgH	flagella structure
KVH_12350	flagella motor protein MotA	motor
KVH_12355	FlhB HrpN YscU SpaS family protein	export apparatus
KVH_12360	flagella biosynthetic protein FliR	export apparatus
KVH_12365	flagella biosynthesis protein FlhA	export apparatus
KVH_12370	lateral flagella motor protein MotA	motor
KVH_12375	flagella basal body-associated protein FliL	flagella structure
KVH_12380	flagella M-ring protein FliF	flagella structure
KVH_12385	putative flagella biosynthesis	unknown
KVH_12390	flagella motor switch proteins FliN and FliM	motor
KVH_12395	flagella biosynthetic protein FliP	export apparatus
KVH_12400	flagella P-ring protein (Basal body P-ring protein)	flagella structure
KVH_12405	flagellin and related hook-associated protein	flagella structure
KVH_12410	flagella hook-associated protein FlgK	flagella structure
KVH_12415	flagella hook protein FlgE	flagella structure
KVH_12420	OmpA/MotB,flagellar motor protein MotB	motor
